# Extract from a Letter to the Baltimore Editor

**Published:** 1843-12

**Authors:** J.C. McCabe


### ARTICLE II.

*Extract from a Letter to the Baltimore Editor.* By DR. J.C. MCCABE.

*Virginia, July* 31, 1843.

DEAR DOCTOR.—I regret that indisposition prevented me from fulfilling, in Baltimore, at the last annual meeting of the "American Society of Dental Surgeons," the appointment made at the previous meeting in Boston—an appointment as flattering as it was unmerited. However, my regret at not being able to be present on the interesting occasion alluded to, is modified by the belief that the distinguished members of the profession, appointed as my coadjutors, acquitted themselves in a manner commensurate with their fame.

It is a matter of congratulation to the worthy of the profession, that through the instrumentality of those, who themselves have arrived at the very summit of professional excellence, our long degraded science has been dragged from the odium that selfish cupidity, and ignorant quackery had thrown around it, and once more placed upon its pedestal, beside sister sciences, whence it had been hurled by hands that touched but to desecrate.

Do not think that it is my design to *lecture* the profession upon any one subject in the range of our science *proper;* older and abler hands have opened to our eyes the volume of their experience, enriched by the successive offerings of successive pilgrims, in the pursuit of professional knowledge, until the cry of "Eureka" has resounded from one chamber of the temple to all the rest. The harvest, which at one period, seemed to be whitening beneath the sun, untouched, has been gathered by the early reapers; and it would seem, now, we are compelled to pause, for there seems to be scarcely a grain left to the late gleaners in the field.

I look then, upon our association, more as a band of union between worthy professors of our art, than as a school for instruction in the science—designed to protect the gentleman, and the scientific practitioner, from the odium that should ever attach to the charlatan—intended "to give the world assurance" of professional competency, and moral worth, and also to hold up to scorn and contempt, the man, who void of a knowledge of his professed science, and wanting in moral stamina, would take advantage of the ignorance, or confidence of the community.

It would not suit the limits of this letter to enter into a comparison, of the various theories set afloat at different periods, by opposing writers on the theory and practice of dental surgery; much error has undoubtedly been mixed with much real good; and as all science in its infancy has had to struggle slowly through the mists which necessarily invested its discovery, it is not to be wondered at, that men of scientifice knowledge, in assigning our art, "a local habitation, and a name," should have erred in some of their views on the subject. It would only be necessary, if this proposition was sought to be controverted, to turn to the history of "the rise and progress," of our older sister, medicine. How slowly did she travel! How halt by the wayside! How medicine, and mountebankry, physic and folly, travelled together. Now empiricism, and now dogmatism; but look at her now, "throned in light," the discoveries of successive ages tending to place her higher and still higher upon her imperial tower!

Age after age bringing its tribute to her stores; name after name of the gifted ones of earth, swelling the train of her votaries: the mighty march of mind pouring in its hosts to her cause, until the science of medicine has become the loftiest pursuit of the proudest intellects, and its benefits extended to the "farthest verge of our green earth."

I say, it would not be in place to discuss the different theories of writers on the science of dental surgery—but I do think it would not be out of place to make some allusion to those night-mares upon our efforts to elevate the profession, viz. *dental quacks.*

Foreigners have been pleased to say of the Americans, that they are the most gullible people on the face of the earth; and although national pride may make us wince under the sneer, I have sometimes thought our sensitiveness on the subject was that of the "galled jade." It may, nevertheless, if properly viewed, be considered as an *amiable weakness,* an excess of good feeling, growing out of a peculiarity of temperament, superinduced probably by the genius of those institutions we are proud to believe peculiar to our land. In America, I speak now more particularly of our southern states, but I am sure it applies with propriety to other sections of the country, he who bears himself with propriety, and *seems* to deserve encouragement, rarely fails of success.

This would be an elysium indeed—a paradise in truth, did the world at the present day, hold no serpents, to wither in their slimy track, the green leaves and bright flowers, the pleasant pathways of our Eden. But, unfortunately, there are bad men in our world, men who are unpossessed of one moral excellence; men, with whom the sanctity of an oath, and the jest of the merry-Andrew, are alike held in respect; men, in whose cranium the organ of acquisitiveness holds prominence; and to such as these, conventional laws are but as "a rope of sand." The morals of a community, and the etiquette of the brothel, hold equal place in their veneration, and such will be found wherever the footsteps of poor fallen humanity have pressed the soil. They are incidental to no particular profession.

The sacred temple finds them kneeling at its altars, and quaffing its cup of communion. The ermine of justice finds the unhallowed pulses of such, beating beneath its folds. The bar finds such, with their unsanctified hands, grasping the glittering bribe. The bed-side of suffering sickness, is darkened by such, too often, who, for the "yellow trash," drug the bowl, and still the pulse; and it will not be presumed, that *our* profession can expect to be exempt from such characters, without *tests of professional and personal worth,* when we behold in other bodies, that have thrown around them *compacts*, tests of *proficiency* at least, such sad evidences, that if

"Some flowers of Eden we still inherit,
The trail of the serpent is over them all."

No man, I am convinced, should ever enter a profession of which he is ashamed. If his pride makes him revolt from the disadvantages under which that profession may place him, he should weigh well the chances before he risks his feelings in the experiment; and yet I have sometimes felt the shame-spot burn upon my cheek, and the heavings of a proud heart swell my bosom, when, in strange communities, (until known,) I have fancied my profession under ban, because some miserable effigy of man, some soulless, shameless, ignorant pretender had preceded me, disgraced his profession, and injured the health of his patients by his abominable malpractices; and under the influence of such feelings, I would gladly have abandoned my calling, and never breathed its name, had I not remembered that "sufferance" was [not] "the badge of all our tribe;" but that all professions had to bear the odium of unworthy members. Perhaps, though, I might again remark, that all the other professions have had tests, by which admission was gained into their fellowship, but *ours*.

Alas, alas! an unlicensed army, a lawless mob, the practitioners of dental surgery have gone forth, "armed to the *teeth*," too literally; flourishing their tooth keys, and in some respects slaying, like Sampson, the Philistines, with the jaw-bone of many an ass, till their names have struck terror to neighborhoods, their practices destroyed many a good constitution, and laid low in death many a beautiful brow, and many a rosy lip; until the profession, Atlas like, has had to bear upon its shoulders a world of obloquy.

The intellectual and the honorable members of the profession, bore the opprobrium, smarting, it is true, under its infliction, until a bold and determined few, men of "stout hearts and strong hands," determined to save their favorite science, or sacrifice in the effort, resolved, for the purpose of protection, and as an evidence to the world of professional integrity, to form "The American Society of Dental Surgeons." Honor to those men! levelling the pretensions of the charlatan, and guarding the public against imposition, gathering the experience, and the wisdom, and the learning of the profession together, for mutual good and public benefit.

This association, then, is one of the *strong tests* of professional excellence; and if, with a knowledge of our society, the public should choose to employ one of whose pretensions they know nothing, they deserve all the evils that may grow out of their folly; because we are pledged by our compact to admit none to our fellowship, to grant our diploma to none, who are not thoroughly qualified to practise in every department of the art. Since an inducement to the really deserving practitioners, (and there are many who as yet have not united with us in our crusade against quackery,) to join with us in our undertaking: and if all who truly deserve well of their community would do thus, the public would then act advisedly in sending from among them, with scorn, the individual who could not furnish, *on demand*, the evidence of his competency, the credentials of his ability.

It is due to the reputation of honorable and high-minded practitioners, that have not united with us, that they *should* do so, and I must confess, when I hear of an individual, an ornament to his profession, objecting to our association, I am painfully compelled to believe his opposition to be the morbid growth of an ill formed jealousy.

Contrast a few years past with the present time, the state of dental practice at the period we are in, to that of twenty-five or thirty years ago; view the strange and conflicting theories of past times—theories indulged in by even the scientific, and compare those expositions, those institutes of practice, with the *rationale* of the profession at the present day, and behold the triumph of our art.

The learned John Hunter could err; the professionally talented Fox could recommend that which the humblest member of our profession, acquainted with his business, would reject; the truly intellectual Bell might now have a lesson on practice read to him; the fancies of many a popular writer, in his day, would now be repudiated as unsound and unworthy: and how have these theories been exploded? By individual effort, truly, but individual effort directed to the enlightenment of the body.

How has dental surgery, stript of all the obsolete and injurious, been elevated to its high state of perfection? I answer, *by effort,* prompted by a noble disposition to rescue a valuable profession from odium; and although it is individual effort, working apart, yet moving a sympathetic chord in the bosoms of the enlightened members of our calling, to reduce to *system,* the principles of our profession, by furnishing a correct theory of practice.

If, without the aid of an association, individual effort has accomplished so much, how must we congratulate ourselves, when we behold the most eminent of our calling, uniting their wisdom and experience, to give tone, and character, and reputation to the science?

